# Dynamic Mechanical Behavior of Hierarchical Resin Honeycomb by 3D Printing

**DOI:** 10.3390/polym13010019

**Published:** 2020-12-23

**Authors:** Huan Hong, Menglei Hu, Liansong Dai

**Affiliations:** State Key Laboratory of Explosion Science and Technology, Beijing Institute of Technology, Beijing 100081, China; hmlbit2017@163.com (M.H.); dailiansong@yahoo.com (L.D.)

**Keywords:** hierarchical honeycomb, 3D printing, collapse strength, energy absorption

## Abstract

In this paper, surface projection micron stereo-lithography technology (PμSL) by 3D printing was used to prepare two resin honeycomb materials with different levels, and the mechanical behavior of these materials was studied. The quasi-static compression experiment and the dynamic compression experiment were carried out on the samples using the in situ micro-compression testing machine and the Split Hopkinson bar (SHPB) experimental equipment. The stress–strain curves of these materials at different strain rates were obtained, and the energy absorption characteristic of materials with two different levels were analyzed. This article reveals that the collapse strength and energy absorption properties of the materials are related to the hierarchical level of honeycomb. Multi-level hierarchical honeycomb (MHH) has higher collapse strength and better energy absorption properties than single-level hierarchical honeycomb (SHH). It turned out that increasing the hierarchical level of honeycomb could improve the mechanical properties of the materials. In the future development of products, the mechanical properties of hierarchical material by 3D printing can be further optimized through changing the level of the fractal structure.

## 1. Introduction

Lightweight and low-density cellular structures are widely used in automobile manufacturing, aerospace and other economic and defense fields. As typical low-density, lightweight cellular structures, honeycombs have excellent mechanical properties and good energy absorption properties [1,2,3]. The cell section of honeycomb materials is usually hexagonal, but it is also triangular, rectangular, or rhombic. The same as foam materials, the mechanical properties of honeycomb materials directly depend on the shape, size, and topology of the cells [4,5,6]. For example, the connection factor of the edge and the surface, the number of adjacent holes, etc., will change with the change from one structure to another and affect the mechanical behavior of the material in an important way. Especially under the impact load, the high frequency component of the load will control the dynamic response of the structure, and the influence of the spatial topology of the cell on the evolution of the local dynamic stress of the material becomes more and more significant. Therefore, in addition to the geometric parameters of the structure, how to establish the relationship between the topological parameters of the cell in the local space and the dynamic response of the material is also an important topic in the description of the mechanical characteristics of honeycomb materials.

Currently, many scholars studied the mechanical properties of honeycomb under different topologies using experimental and numerical simulation methods [7,8,9,10]. Hohe et al. [5,6] studied the elastic response of hexagonal, triangular, and quadrilateral honeycomb structures. Zhen et al. [11] numerically studied the dynamic mechanical properties of irregular random honeycomb structures under in-plane impact. Yamashita [12] analyzed the impact behavior of different cell honeycomb specifications with numerical and experimental methods. Zinno et al. [13] analyzed the multi-scale approach for the design of composite sandwich structures for train application. Wu and Jiang [14] studied the experimental results of six honeycomb structures under quasi-static load and axial load. Reza et al. [15] studied the in-plane compression mechanical properties of hexagonal honeycomb based on additive manufacturing technology by comparing experimental and calculated results. Different cell configurations [16,17,18,19,20,21] have been developed and explored over the years. To determine the relationship between compressive strength and cell size, some studies [22,23,24,25] found that changing some cellular size parameters could increase the compressive strength of the structure, thereby improving the mechanical properties of the honeycomb structures. Anna et al. [26] studied the energy absorption of honeycomb with regular cellular structures by 3D printing through static and dynamic loading. Many studies have shown that the mechanical and energy absorption properties of honeycomb largely rely on its cellular configuration and base material’s properties. At present, based on the printing of existing material models and the construction of composite materials, 3D printing has seen some new applications. Kozior et al. [27] studied to improve the mechanical stability of an electrospinning nanofiber mat, which was combined with 3D printing technology to prepare a mechanically stable filter with a nanofiber surface. This study showed that the nanofiber mat had a strong adhesion to 3D printed scaffolds from thermoplastic polyurethane (TPU). At the same time, the adhesion of three-dimensional printing on textile fabrics [28] described the improvement of textile/polymer adhesion based on experimental research on adhesion in 3D printed textile/polymer composites and theoretical research on adhesion inside the 3D printed part.

A large amount of research currently focused on the change of the geometric parameters of the cell structure, while relatively little research has been done on multi-level hierarchical honeycomb structure designed by biologically inspired principles [29], which has seriously hindered its application in aerospace, rail, and automobile industry. Recently, the rapid development of 3D printing technology has met the design requirements of complex three-dimensional spatial structures, and it has provided a more convenient way for the preparation of porous composite materials and the optimization of fine–macro cross-scale [30]. In this paper, multi-level hierarchical honeycombs and single-level hierarchical honeycombs were fabricated by additive manufacturing to research their impact on mechanical behavior. The impact yield strength and energy absorption properties of these materials were investigated. 

## 2. Experimental Process

### 2.1. In Situ Micro-Compression Experimental Process

**Figure 1:**
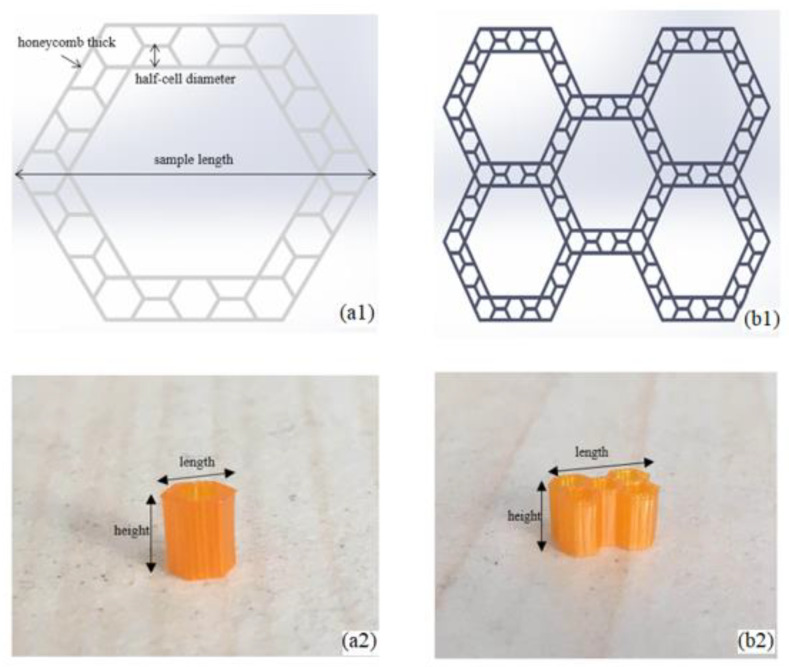
(**a**) Single-level hierarchical honeycomb (SHH), (**b**) multi-level hierarchical honeycomb (MHH).

**Figure 2:**
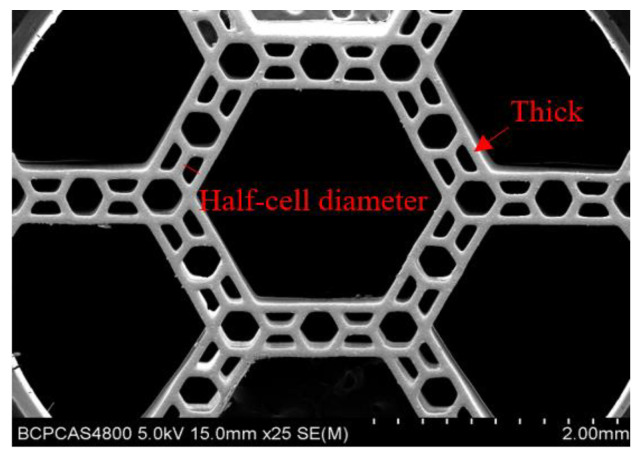
Multi-level honeycomb under SEM.

**Figure 3:**
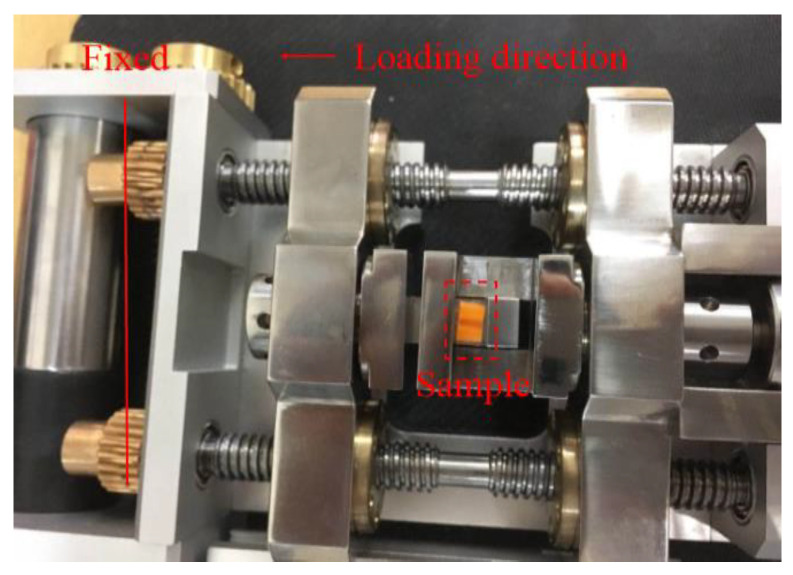
The in situ compression test equipment.

In this study, two different samples (single-level hierarchical honeycomb (SHH) with a size of 4 mm × 2.6 mm and multi-level hierarchical honeycomb (MHH) with a size of 4 mm × 6 mm) were fabricated as shown in Figure 1a,b. The resin honeycombs were manufactured by surface projection micron stereo-lithography technology (PμSL) technology (BMF Inc., Shenzhen, China) that meets the stereo lithography apparatus (SLA) technical standards in ISO/ASTM 52921 and had density in the range of 0.347–0.372 g/cm^3^ (the parameters of resin listed in Table 1 and Table 2), meaning a half-cell diameter of 0.14 mm and resin honeycomb 0.07 mm thick (see Figure 2). In view of the millimeter size of the samples, the in situ micro-compression loading test was performed by a MICROTEST2000 system (GATAN Inc., Pleasanton, CA, USA) with SEM (Figure 3). The system comprised a replaceable load sensor, a sample heater, a clamping device, and a compression head, and it is an important platform to observe and analyze the micro-deformation morphology and fracture mechanism of materials. The honeycomb samples were clamped between two compression heads with a dimension vision of 1 mm × 1 mm, such that an aperture of 0.28 mm diameter [31] was exposed in the vision (Figures 8 and 9). We respectively observed the deformation of the honeycombs and the deformation of the apertures by axial compression and radial compression. Before testing, the upper compression head was given a fixed speed and the samples were fixed on the base. While observing the deformation of the samples by SEM, the information of the loading was recorded.

### 2.2. Dynamic Experimental Process

**Figure 4:**
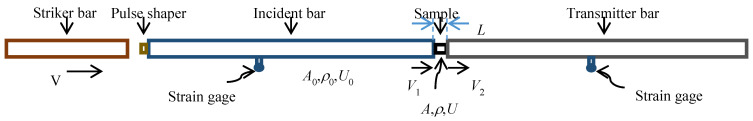
Schematic diagram of split Hopkinson pressure bar.

**Figure 5:**
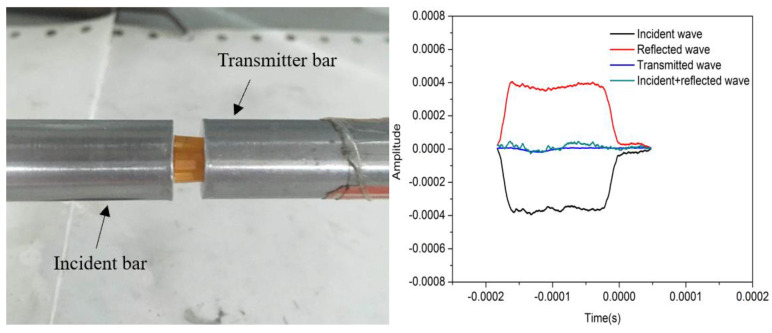
Experimental process and pulse signal by SHPB.

The Hopkinson pressure bar can realize loading experiments under a high strain rate (10^2^–10^4^ s^−1^), and it is widely used by scholars engaged in dynamic mechanical research and related fields [32,33,34]. The core parts of the Hopkinson pressure bar device are two separate elastic waveguide bars (the incident and the transmitted bar). The sample is clamped between the two bars. The loading pulse is generated by the striker bar hitting the incident bar. The device is shown in Figure 4. An incident stress wave with a wavelength twice the length of the striker bar will be generated in the incident bar. When the incident stress wave reaches the sample, part of it is reflected back to the incident bar, and other part passes through the sample and reaches the transmitter bar. The transmitted stress wave is captured by the absorption bar and finally absorbed by the energy harvester. As shown in Figure 5, the signals of the incident wave, the reflected wave, and the transmitted wave have three amplitudes *ε_I_*, *ε_R_*, and *ε_T_*, respectively. From these three signal values, we can deduce the stress–strain relationship of the sample [35]. 

According to the assumption of a one-dimensional stress wave in the bar, the stress σ, strain ε, and strain rate $\dot{\varepsilon }$ of the sample can be calculated by the following equations:

(1) $$\sigma (t)=E_{0}\frac{A_{0}}{A}\varepsilon_{T}(t)$$

(2) $$\dot{\varepsilon }(t)=-\frac{2C_{0}}{L}\varepsilon_{R}$$

(3) $$\varepsilon (t)=-\frac{2C_{0}}{L}\int_{0}^{t}\varepsilon_{R}dt$$

Here, *E*_0_ is the elastic modulus of the bar, *A*_0_ is the cross-sectional area of the bar, *A* is the cross-sectional area of the sample, *C*_0_ is the elastic wave velocity of the bar, *L* is the length of the sample, $\varepsilon_{T}(t)$ is the transmission strain, and $\varepsilon_{R}$ is the reflected strain.

In this paper, a 14 mm aluminum SHPB loading system was used to carry out an impact test, and the dynamic compression performance of the hierarchical material prepared by 3D printing in the strain rate range of 500–1500 s^−1^ was studied. In these experiments, the diameter of the striker bar and all the bars were 14 mm, the length of the striker bar was 400 mm, and the length of the incident bar and transmitted bar were both 2000 mm. A set of strain gauges was attached between the incident bar and the transmitted bar, and each group has two strain gauges placed on the opposite surface of the bar to account for the transverse inertia effect of the stress wave. Taking into account the low impedance characteristics of the hierarchical materials and the attenuation effect of the bar on the stress wave, a semiconductor strain gauge was used on the transmitted bar, and a resistance strain gauge was attached to the incident bar. Each set of strain gauges was connected to a dynamic strain gauge to process and amplify the signal. Then, the incident pulse, reflected pulse, and transmitted pulse were collected by an oscilloscope.

## 3. Results and Discussions

The honeycomb samples were loading at low speed (MHH with the speed of 0.24 and 0.18 mm/min (the corresponding strain rate is 10^−3^ s^−1^) respectively in axial and radial compression loading, SHH with the speed of 0.24 and 0.3 mm/min (the corresponding strain rate is 10^−3^ s^−1^) respectively in axial and radial loading). The sample was clamped between two rigid indenters (see Figure 3). When loading, the right indenter moves to the left to compress the test piece, while the left indenter remains stationary. Before loading, we applied petroleum jelly to the surface of the left and right indenters to reduce the friction between the test piece and the indenter interface. All samples were loaded to compactness, the corresponding loading force and displacement could be obtained from the force at the right indenter and the displacement sensor, and the nominal stress–strain curve of the material could be obtained by calculation. For each group of working conditions, the test results with better repeatability were taken as the final test results.

**Figure 6:**
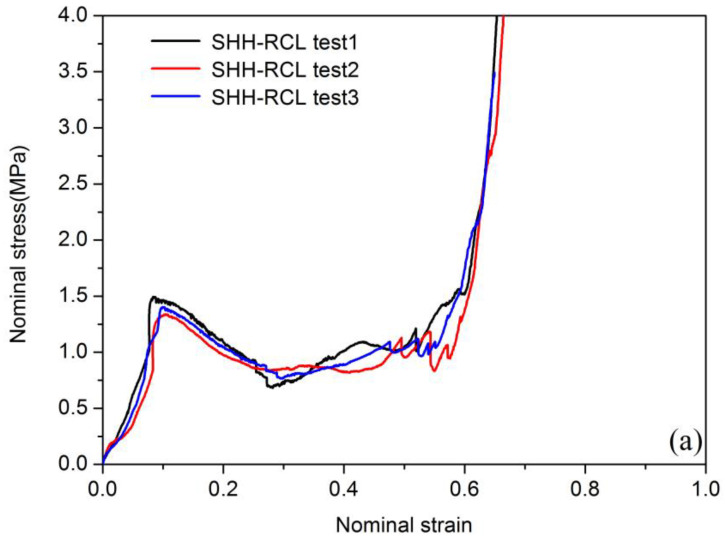

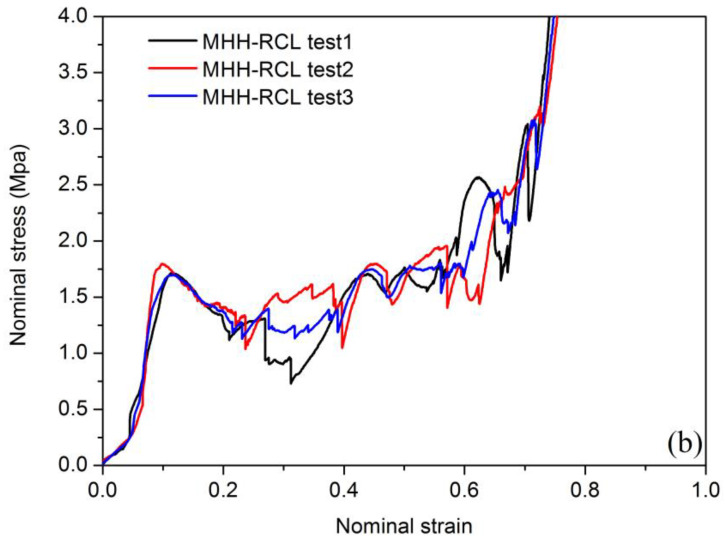
Nominal stress–strain curve of radial compression loading (RCL): (**a**) SHH-RCL test, (**b**) MHH-RCL test.

**Figure 7:**
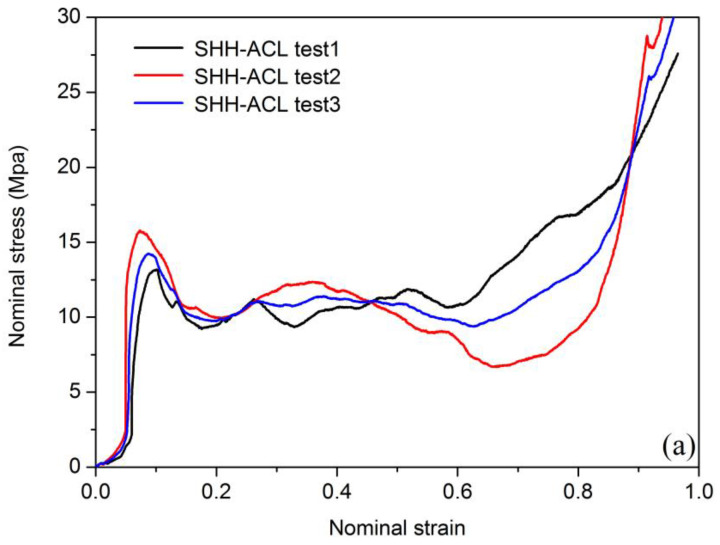

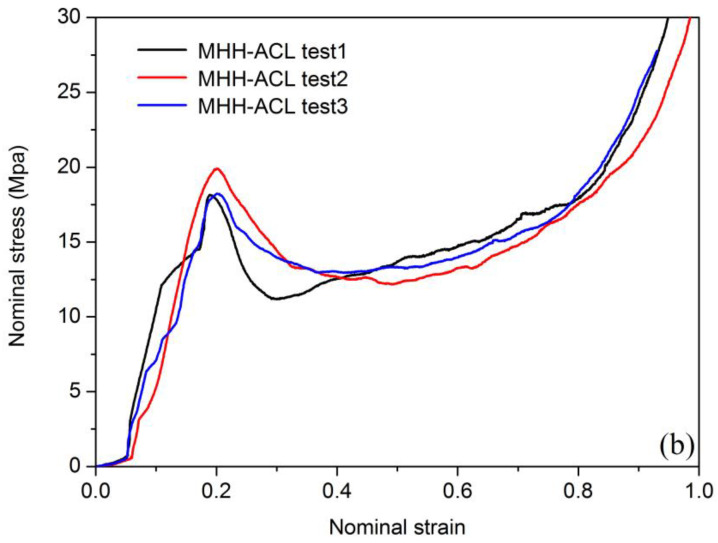
Nominal stress–strain curve of axial compression loading: (ACL) (**a**) SHH-ACL test, (**b**) MHH-ACL test.

Figure 6 and Figure 7 show nominal stress–strain curves in quasi-static loading by an in situ micro-compression experiment. As it can be seen, very good reproducibility between curves was achieved. The stress–strain curves of the two levels of hierarchical materials show the same pattern in the same loading direction, and they can be divided into three stages: initial linear elastic section, stress plateau section, and stress compaction section. In the initial elastic region, the cell walls of the hierarchical materials enter the elastic deformation stage, and the stress–strain of the material shows a linear relationship; as the loading progresses, the cell wall begins to yield and gradually collapses, and the stress enters the platform section; finally, when all the cell walls are compacted, the stress curve rises rapidly during the compaction section. It is worth noting that there will be a sharp drop when the stress reaches the first peak, which is due to the internal cell wall of the material beginning to fail and fracture and collapse. When the remaining intact cell wall structure begins to load, the stress will rise again into the platform section. Different from the axially compressed long and smooth platform section, the stress platform section of the SHH material exhibits obvious oscillations, mainly due to the poor toughness of the base honeycomb material. The curve that appears in the elastic stage of individual materials is not smooth, and the difference is mainly related to the micro-defects of 3D printing materials.

The stress–strain curves of two honeycombs radial compressions are described in Figure 6. As shown in Figure 6a, stress rises slowly with the increase of strain. After the stress reaches the peak value of 1.49 MPa, the curve shows a stable plateau stage. A similar situation also occurs in Figure 6b. As shown in Figure 6b, the stress–strain curve peak value reached 1.75 MPa, which is significantly higher than that in Figure 6a. This shows that the radial compression loading peak value of MHH is 17.4% higher than SHH. For axial compression loading, the peaks of the curves of the two honeycombs are higher than those of the radial compression loading, which can be seen in Figure 7a,b. As shown in Figure 7a, the peaks of curves reached 15.79 MPa, and in Figure 7b, the peaks of curves reached 20.03 MPa. The axial compression loading peak value of MHH is 21.2% higher than SHH. The results show that the compressive capacity of MHH is better than SHH. Compared with the research results of Xu et al. [36], it is found that the quasi-static collapse strength of the hierarchical honeycomb structure is significantly higher than that of the ordinary honeycomb.

**Figure 8:**
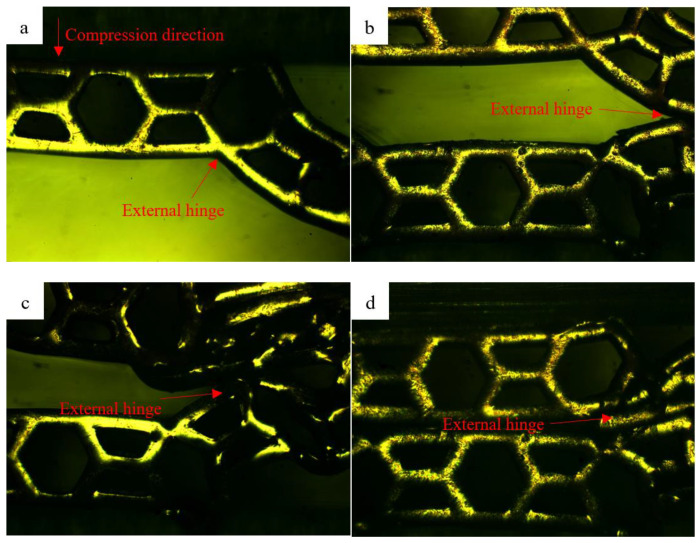
SHH material overall deformation process. (**a**) ε = 0.12, (**b**) ε = 0.29, (**c**) ε = 0.38, (**d**) ε = 0.56.

**Figure 9:**
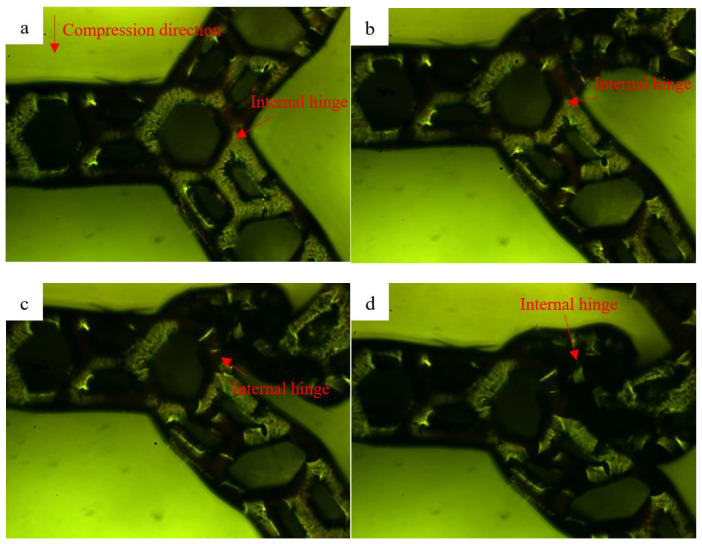
MHH material local hinge deformation process. (**a**) ε = 0.08, (**b**) ε = 0.23, (**c**) = 0.36, (**d**) ε = 0.52.

In order to visually observe the compression deformation process of the multi-level honeycomb, we selected the deformation process perpendicular to the displacement direction of the vision. Figure 8 and Figure 9 show the SHH material overall deformation process and MHH material local hinge deformation process, respectively. As shown in Figure 8, the first that started was the distortion of SHH external hinge. As the compression increased further, the honeycomb holes of the external hinge were twisted into clusters. When the crushing was over, the honeycomb holes on the upper and lower contacting with surfaces of the indenter would be distorted. As shown in Figure 9, the honeycomb hinge parallel to the surface of the indenter does not undergo significant deformation. Conversely, the angled internal hinges of honeycomb on both sides appear to be deformed as the crushing progresses and finally twisted into clusters.

Comparing the compression process in two directions, the load-carrying capacity and compression resistance exhibited by the axial compression process are better than the radial compression process. Therefore, in the future development of products, we should focus on the study of axial compression capability. Comparing the collapse strength of the two compression modes, it is not difficult to see that the collapse strength of the MHH material is obviously higher than that of the SHH material. Therefore, the mechanical properties of the MHH material can be further optimized by increasing the level of topological stages.

**Figure 10:**
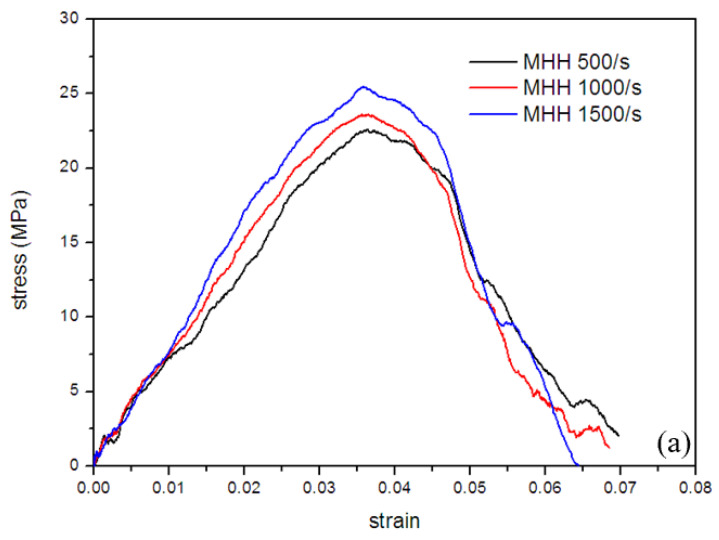

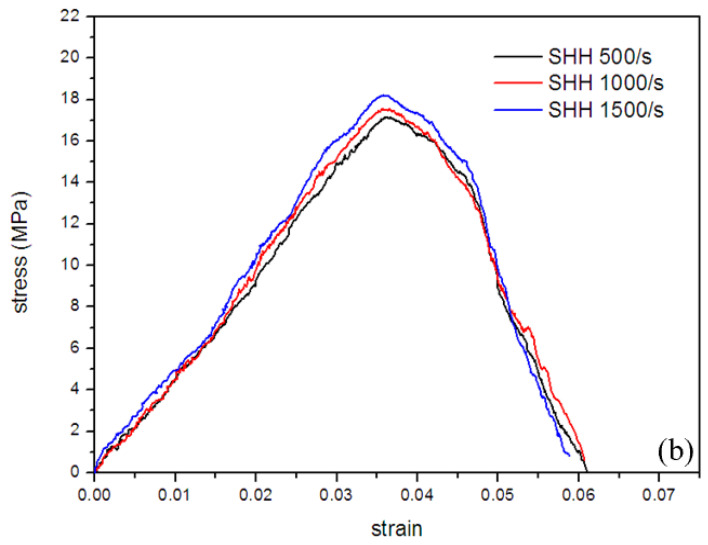
Dynamic stress–strain curve of resin hierarchical honeycomb materials: (**a**) MHH, (**b**) SHH.

In the SHPB impact test, the bullet speed range is 2–6 m/s, and the corresponding load strain rate range is 500–1500 s^−1^. Figure 10a,b show the dynamic stress–strain curves of the two resin honeycomb materials with good repeatability under different strain rates. It can be seen from the figure that the dynamic stress–strain curve of the material is different from the quasi-static stress–strain curve. It has experienced an elastic stage before the stress reaches the initial peak, and then the stress begins to drop sharply due to the failure of the cell wall. All samples are compacted. Although the impact loading speed is low, due to the small size of the sample, the matrix resin material exhibits low toughness under dynamic loading, resulting that the dynamic stress–strain curve of the honeycomb specimens does not increase as the stress does under quasi-static loading. The platform stage is different from common metal porous materials. It can be seen from the figure that the two hierarchical materials of the unit cell size exhibit different strain rate sensitivity. Under the three strain rates studied, the stress–strain curve of the SHH material is basically similar, while the MHH material shows a certain strain rate sensitivity, and the collapse strength of the material increases at a high strain rate. Sahu [37] and Onck [38] pointed out that the larger the pore size of the foam material, the lower its strength. It can be seen from Figure 10 that although the MHH material has the same pore size as the SHH material, it has a lower relative density, and its collapse strength at different strain rates is higher than that of the SHH material. The above research shows that the reason for this difference may be related to the level number of honeycomb materials. The more levels honeycomb has, the higher the collapse strength.

**Figure 11:**
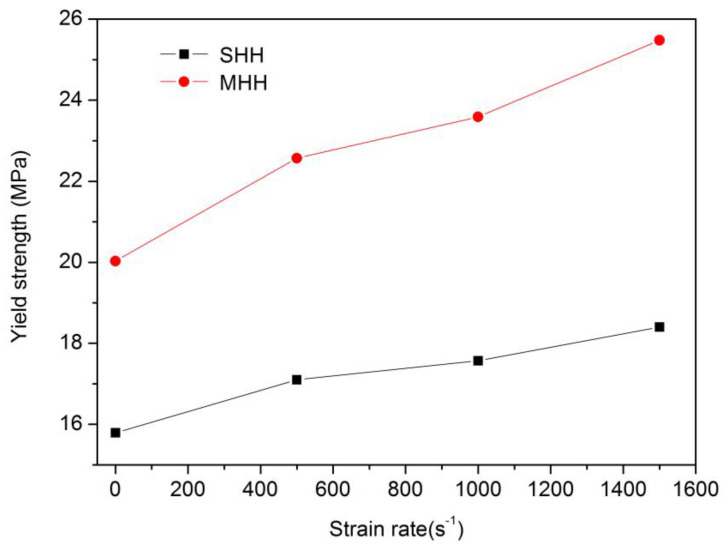
The yield strength of resin hierarchical honeycombs under different strain rates.

In the past, many scholars discussed the strain rate sensitivity of porous structures. Zheng et al. [39] pointed out that due to the change of material deformation, the strength of porous medium under high-speed impact loading is higher than that under quasi-static loading. However, under low-speed impact loading conditions, the strengthening mechanism of material strength is still unclear. Figure 11 and Table 3 show the collapse strength of two hierarchical honeycomb materials with different levels under different strain rates. It can be seen that the two honeycomb samples exhibit different strain rate sensitivity. As shown in MHH materials, when the strain rate increases from 0.001 s^−1^ to 1000 s^−1^ and 1500 s^−1^, the collapse strength increases by 17.78% and 27.21%, respectively. However, as shown in SHH materials, the strain rate strengthening effect is not obvious. The material collapse strength only increases by 16.53% when the strain rate increases from 0.001 to 1500 s^−1^.

The different strain rate sensitivity of the two hierarchical honeycomb materials may be related to the difference in the micro-structure of the materials. Since the optimal preparation process parameters have not yet been determined, the quality of the single cell wall inside the honeycomb material is poor, and the cross-sectional dimension is not constant (see Figure 2). The micro-defects of the hole wall structure may reduce the strain rate strengthening effect of the material strength. Another reason that affects the strain rate sensitivity of materials may be related to the difference in the levels of honeycomb. The more levels the honeycomb has, the more obvious the strain rate strengthening effect. Due to the limited number of transverse unit cells of SHH material, the sample lacks transverse hinge constraints during impact loading, which aggravates the deformation and failure of the material, thereby reducing its strain rate sensitivity.

The honeycomb porous material is a typical buffering energy-absorbing protective material, so the energy absorption properties of resin hierarchical honeycomb materials under different strain rates are considered in this article. The work done by the external force is continuously folded through the honeycomb wall and converted into the shaping energy required for honeycomb deformation. The energy absorbed per unit volume of the honeycomb (*W*) can be characterized by the area surrounded by the stress–strain curve, and the equation is as follows:

(4) $$W=\int_{0}^{\varepsilon_{p}}\sigma (\varepsilon )d\varepsilon$$

Here, *ε_p_* is the strain of compressed before the densification phase, *σ* is compressive stress, and *ε* is compressive strain.

As an ideal energy absorbing structure, the ratio of the energy absorbed by the unit volume in the densification unit to the platform stress is an important parameter for describing the energy absorption characteristics. The expression equation for energy absorption efficiency (η) is:

(5) $$\eta =\frac{\int_{0}^{\varepsilon_{p}}\sigma (\varepsilon )d\varepsilon }{\sigma_{\max}\cdot \varepsilon }.$$

Here, *ε_p_* is the strain of compressed before the densification phase, *σ_max_* is maximum stress on a given strain path, *σ* is compressive stress, and *ε* is compressive strain.

**Figure 12:**
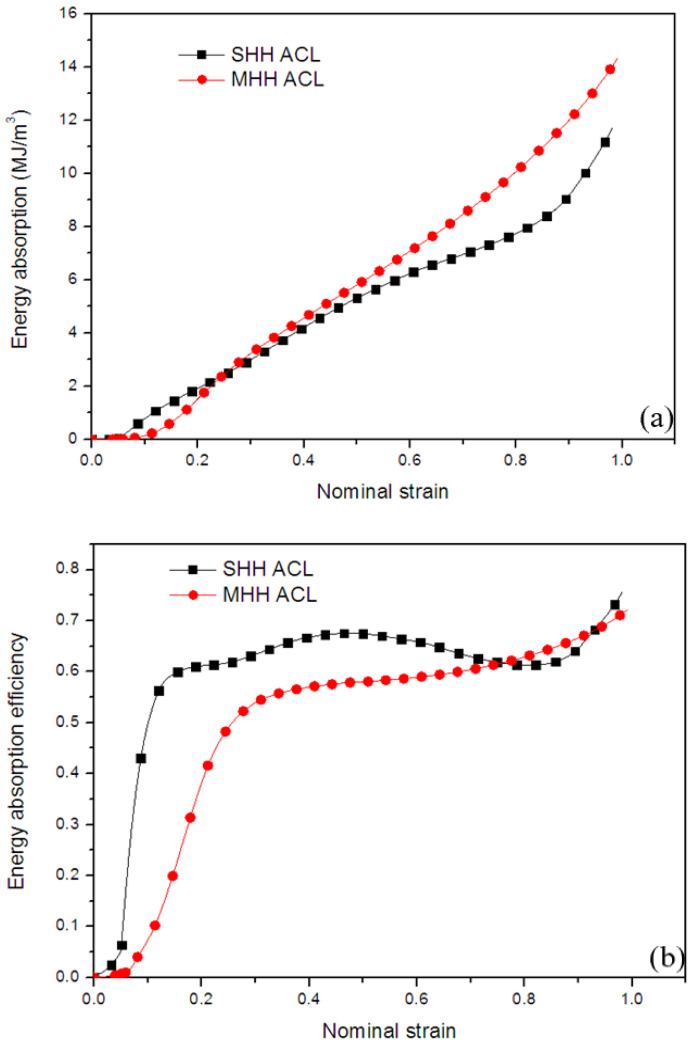
Energy absorption properties of resin honeycomb material under quasi-static loading: (**a**) Energy absorption, (**b**) Energy absorption efficiency.

**Figure 13:**
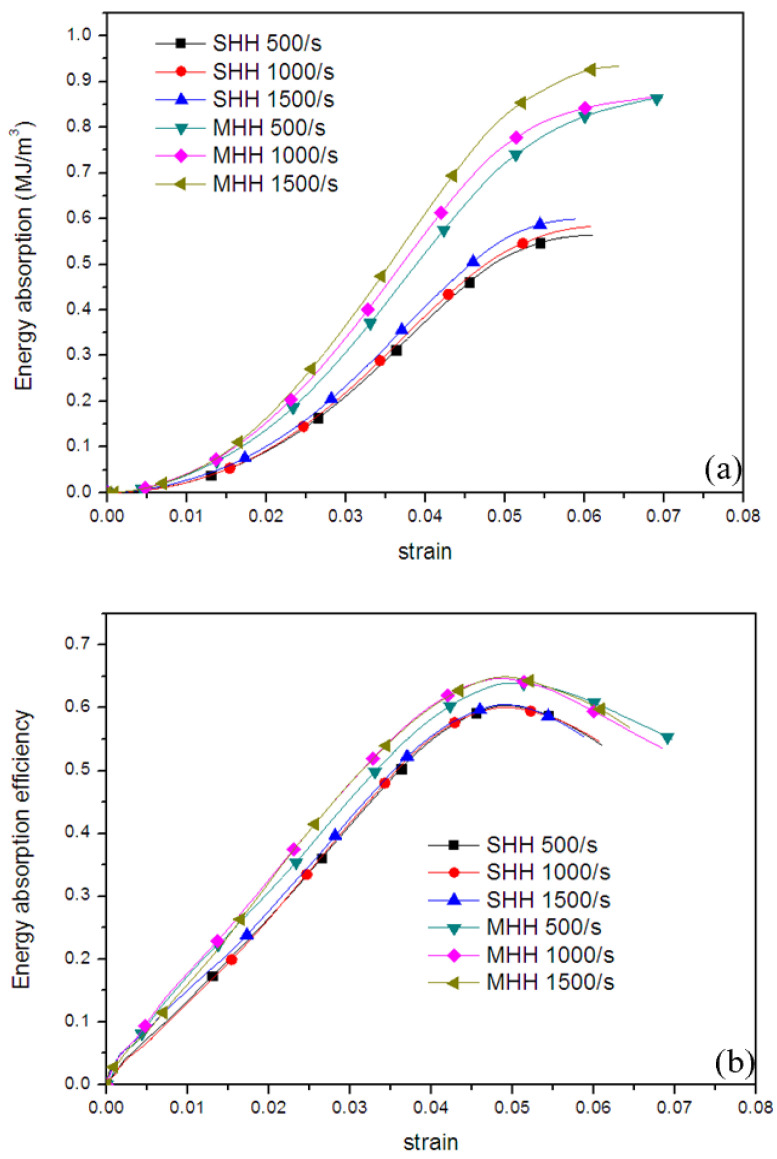
Energy absorption properties of resin honeycomb materials under dynamic loading of different strain rates: (**a**) Energy absorption, (**b**) Energy absorption efficiency.

Figure 12 and Figure 13 respectively show the energy absorption and energy absorption efficiency of resin honeycomb porous materials with different levels under quasi-static and dynamic loading. It can be seen from Figure 12a that under quasi-static loading, the energy absorption of the MHH material is slightly higher than that of the SHH material. However, it is worth noting that although the MHH material absorbs more energy, it cannot guarantee higher energy absorption efficiency. The main reason for this phenomenon is that the MHH material has higher stress and dense strain. It can be seen from Figure 13a that as the strain rate increases, the strain rate strengthening effect of the MHH material is more obvious, and the energy absorption of MHH is significantly higher than that of the SHH material. The comparison of energy absorption efficiency in Figure 13b shows that the energy absorption efficiency curve of resin honeycomb material is similar to that of foamed titanium [40], with obvious peaks, and the energy absorption efficiency of the material increases as the strain rate increases. Under dynamic loading, although the collapse strength of the material increases with the increasing of the strain rate, the low toughness of the material under dynamic loading causes the stress of the honeycomb material to decrease significantly after reaching the initial peak value, thereby reducing its energy absorption capacity. In short, whether it is quasi-static or dynamic loading, the energy absorption capacity of the MHH materials is significantly better.

## 4. Conclusions

The paper presented the mechanical properties of hierarchical resin honeycombs. With the goal of light energy absorption of materials, the collapse strength and energy absorption efficiency of honeycombs with different levels under quasi-static and dynamic loading conditions are analyzed and compared. Based on the results obtained, the following can be stated:

(1) Under quasi-static loading, by comparing the experimental curves of the two compression directions, we find that the axial compression bearing capacity of the sample is higher than the radial compression. In future research, we should focus on the axial compression capability of honeycomb.
(2) Both under quasi-static and dynamic loading conditions, the collapse strength of the MHH materials is higher than that of the SHH materials. The mechanical properties of honeycomb materials can be improved by increasing the level of honeycomb cells. Under dynamic loading, the initial collapse strength of the honeycomb material shows a certain strain rate sensitivity, and the strain rate effect of the MHH material is more obvious. The two honeycomb materials with different levels show different strain rate sensitivity, which may be related to the difference of the material microstructure.
(3) As the strain rate increases, the energy absorption and energy absorption efficiency will also increase. In general, the MHH material has more energy absorption than the SHH material.

## Figures and Tables

**Table 1:** The parameters of resin material provided by manufacturers.

Resin Type	Tensile Strength (MPa)	Impact Strength (J/m)	Elongation at Break (%)	Viscosity (CPs @ 25 °C)	Hardness
Tough	50–60	35–50	8–12	180–280	75–80 (ShoreD)

**Table 2:** The parameters of resin honeycombs.

Material	Sample Length (mm)	Sample Height (mm)	Honeycomb Thick (mm)	Half–Cell Diameter (mm)	Sample Weight (g)	Relative Density (g/cm^3^)
SHH	2.6	4	0.07	0.14	0.010	0.369
MHH	6	4	0.07	0.14	0.049	0.347

**Table 3:** The yield strength of resin honeycombs under different strain rates.

Style of Honeycomb	Strain Rate/s^−1^	Yield Strength/MPa
SHH	0.001	15.79
	500	17.1
	1000	17.57
	1500	18.4
MHH	0.001	20.03
	500	22.57
	1000	23.59
	1500	25.48
